# Rare upside of climate-induced phenological changes: Gentoo penguins *(Pygoscelis papua)* avoid heat events at Martillo Isl., Tierra del Fuego, Argentina

**DOI:** 10.1371/journal.pone.0347877

**Published:** 2026-05-20

**Authors:** Sabrina Harris, Ignacio Juárez Martínez, Tom Hart, Andrea Raya Rey

**Affiliations:** 1 Laboratorio de Ecología y Conservación de Vida Silvestre, Centro Austral de Investigaciones Científicas (CADIC-CONICET), Ushuaia, Tierra del Fuego, Argentina; 2 Wildlife Conservation Society Representación Argentina, CABA, Buenos Aires, Argentina; 3 School of Biological and Medical Sciences, Oxford Brookes University, Headington Campus, Oxford, United Kingdom; 4 Instituto de Ciencias Polares, Ambiente y Recursos Naturales (ICPA), Universidad Nacional de Tierra del Fuego de Antártida e Islas del Atlántico Sur, Ushuaia, Tierra del Fuego, Argentina; Universita degli Studi di Bari Aldo Moro, ITALY

## Abstract

Heat waves have detrimental effects on most species, and particularly on cold-adapted species such as penguins. In this study we aim to understand what are the responses to heat weather events of Gentoo penguins (*Pygoscelis papua*) at one of their northernmost colonies: Martillo Island in Argentina. This colony was monitored using a time-lapse camera between 2013 and 2024. In this study we observe acute heat-related mortality events such as the death of five penguin chicks within forty-five minutes while recorded temperatures reached 24°C. During the observation period we found Gentoos are advancing their breeding season by 2 days per year, which is concurrently reducing the number of days exposed to high temperatures (≥ 20°C). While future climate change scenarios may increase the frequency and intensity of heat waves, for now, this is a rare example in which a warming-induced phenological change withdraws chicks from potentially deadly hot summer days by fledging sooner.

## Introduction

Climate change is one of the biggest threats to biodiversity on the planet [1–4]. Temperature rise and the increase in frequency and intensity of heat waves, are some of the consequences which have detrimental effects on most species, either through direct or indirect effects [5]. Nowhere is the potential for direct impact greater than in the polar and sub-polar regions, where adaptations against heat loss leave animals vulnerable to hyperthermia [6–7]. Particularly at the edges of the distribution of a given species, individuals are closer to the limit of tolerance to weather events [8]. Therefore, understanding the devastating effects of these heat events may help assess the vulnerability of species in future climatic conditions.

The detrimental effects of atmospheric and oceanic heat on seabirds have been studied in both subpolar [9–15] and polar regions [16–18]. Alongside climate change predictions, reports of detrimental effects of extreme ambient heat events on seabird survival have increased over the last years [19–27]. Holt & Boersma (2022) [19] observed that recently fed Magellanic penguin (*Spheniscus magellanicus*) chicks were more vulnerable to heat stress than unfed chicks, as food digestion increases metabolism and heat production and may be the tipping point for chicks to succumb to heat. Chicks must feed to grow, particularly during their initial growth period (< 55 days old, [28]) and are therefore more susceptible to hyperthermia. In addition, the creation of feathers is another metabolically active stage that chicks must undergo before fledging, and molting generates excess heat that penguins must dissipate mainly through bill, flippers and feet [29]. If ambient temperatures rise above the thermoneutral range of the species during these metabolically active periods, hyperthermia is more likely to occur.

A species’ potential to adapt to changing environmental scenarios is linked to their adaptability to a given thermal range and their ability to either shift the timing of their breeding cycle to coincide with more favorable conditions, if prey availability is not a limiting factor, or move to other locations [30,31]. Species with broader latitudinal ranges are expected to be more able to adapt by moving to milder and more oceanographic ranges [32]. However, populations at the edges of their distribution, such as northern colonies of penguins, are more exposed to edge-of-range effects, in this case heat events [19,33]. A shift in breeding date has been described for many species, including penguins [34–37]. Some species can shift the beginning of their breeding season to coincide with more favorable conditions [38]. For example, Gentoo penguins in Antarctica breed earlier in seasons with decreased sea ice extent which is more favorable for egg laying [34,39–41].

Non-burrowing adult penguins present two main behavioral adaptations to hot temperatures [42]. To avoid overheating individuals lay down and stretch their flippers and feet out to dissipate heat though increased peripheral blood circulation and irradiation [43]. In addition, they raise their head and pant to increase hot air exchange through increased respiration [44]. Physiological mechanisms may also be at play to protect the body from hyperthermia damage. In some Antarctic species upregulation of heat-shock proteins in warmer regions may help protect the physiological integrity of individuals [16]. Despite their adaptations, temperature increases may still take a toll on immune function as heat-stress induced corticosterone boost has a negative effect on lymphocyte production and egg quality [45,46].

In the case of chicks, their body is covered in down which acts as an insulator from external temperature [47]. This down protects chicks from hypothermia as core temperature is maintained even in freezing temperatures, but excess heat cannot be released and only feet heat exchange or panting can help to thermoregulate. Given the increased susceptibility to weather events of surface breeding penguins, as opposed to borrow digging species, other behavioral changes are due to maintaining breeding performance regardless of climate variability.

Gentoo penguins are ideal study subjects to evaluate the potential impact of local environmental conditions, and extreme weather events in particular, on chick behavior and survival. These penguins present high behavioral plasticity and can modify their laying date to begin earlier in warmer years [48–52]. In addition, given that they are year-round residents at their colony they can adjust their breeding to local environmental conditions, such as warming weather or the timing of the last snowfall of the season [41,53]. Gentoo penguins living in the northern edge of their distribution are exposed to higher temperatures and extreme heat events than those in the South. The objective of the present work is to understand the effects caused by ambient heat, and extreme heat events in particular, on Gentoo penguin chick behavior and survival at one of the northern colonies at Martillo Island, within the Beagle channel, Argentina. Given that breeding is happening sooner for this and other penguin species [52], post-guard chicks could reduce their exposure to hot summer days over successive years.

## Methods

**Fig 1:**
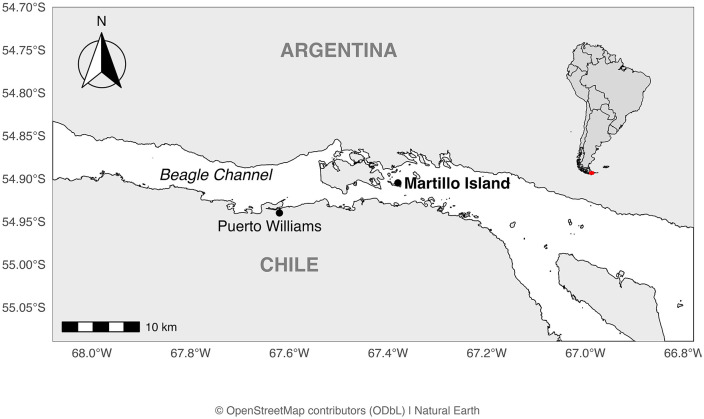
Martillo Island within the Beagle Channel, Argentina. Martillo Island (black dot) in Argentina, and Puerto Willams (black dot) in Chile, shown in map. Insert (top right) shows a scaled map of Argentina and Chile with enlarged area indicated in red. Enlarged map generated with High-resolution Coastline (GSHHS) [57], and smaller map generated with naturalearth package in R [64].

Studies took place at Martillo Isl. (54° 54’ S; 67º 23’ W, Fig 1), within the Beagle Channel, Tierra del Fuego, Argentina, where a small colony of Gentoo penguins recently settled and coinhabits with a Magellanic penguin colony [54–56]. The Gentoo penguin colony has been studied for over thirty years with the population recorded since 1992. This study was evaluated and approved by the Secretaria de Ambiente de la Provincia de Tierra del Fuego, Resol. SUB.P.A. y S Nº043/2017 “Trophic and genetic ecology of seabird assemblages from the Beagle Channel and Staten Island: spatial and temporal variation”. A previous study based on on-the-ground counts showed there was an increase of 18.8% in population size between 1992 and 2011 [55].

### Data collection

In 2013 a Reconyx HC600 Hyperfire trail camera (Reconyx, Inc., Holmen, WI, USA) was placed at 50 cm from the ground inside a hollowed tree trunk which provided a wooden housing that prevented the camera from exposure to direct sunlight, wind, rain and snow. In addition, the camera was facing south to avoid direct sunlight which could interfere with image quality and temperature readings. The camera was placed facing the whole colony (30–60 nests) and has recorded images at one photo per hour (between 0600h and 2100h most years, see S1 Table). Full breeding seasons (September to March) were recorded except for 2017 and 2018 seasons for which information was only obtained until October (S1 Table). Information on presence and timing was recorded according to Black (2017) [58]. In 2013 phenology of the species was described at Martillo Island, the breeding season began in September, and chicks fledged in February, after molt [56]. From now on the season will be named after the year breeding began.

### Nest and chick counts

The following definitions were used to interpret the photographs: “laying” stage was considered to have begun as from the first day individuals were on the nest laying ventrally to cover nest content and did not uncover the nest from that day on. Number of nests were counted over several days within this period (October-December) and the maximum number of nests was used as our estimate of breeding pairs. “Post-guard” start was defined as the moment chicks were not covered completely by the parents while at the nest and were left alone at times. During the post-guard period, chicks gradually lose their down and post-guard ends once their new feathers grow in completely and they are ready to go to sea and are no longer seen at the colony (following [58]). Number of chicks was counted over several days (7–14 days) within post-guard (January-February) and the maximum number of chicks was used to estimate chick survival for each year. In addition, it was noted if chicks were panting (beak open and not in proximity to an adult to avoid confusing with begging). Chicks were deemed dead if they became motionless and no longer moved the remaining days of the season or until carcasses were predated or individuals were removed for necropsies.

### Annual counts of nests and chicks

Between 2013 and 2023, nest numbers increased by an average of 12.2% per year and chicks increased by 12.7% per year. Number of nests and chicks have grown continuously over the study period, with a total of 61 nests and 58 surviving chicks counted in 2023 (Poisson GLM: β = 0.09 ± 0.02 nests/year, z = 5.5 P < 0.001,with year explaining 94% of deviance for nests, and Poisson GLM: β = 0.10 ± 0.02 chicks/year z = 5.6 P < 0.001, with year explaining 85% of deviance for chicks). The average number of chicks per nest was 0.9, ranging between 0.7 and 1.0 (similar to Dodino et al. 2018 [56]). Given that some chicks are likely out of sight in a given frame, we do not consider this parameter as an estimation of breeding success. However, the values fall within the values of breeding success observed for the species at other colonies (e.g., Signy Isl.: 0.05–1.27, [59]; Goudier Isl.: 0.2–1.4, [60]; Falklands/Malvinas Isl.: 0.51–1.44, [61]; Cape Funes: 0.74–1.23, [62]). Duration of breeding season was estimated as the number of days between the beginning of laying and the end of post-guard. A linear model was generated to evaluate the day of the year post-guard ended as a function of year (S2 Table).

### Temperature threshold

Temperatures recorded by the camera were extracted from the photographs as they are recorded by the time-lapse camera. Maximum temperatures were usually around 1300-1500h so we were certain any extreme temperature events were recorded within our study schedule (0600h - 2100h). We established 18ºC as the threshold to mark for extreme temperatures as it is the threshold above the 90^th^ percentile of the historical average maximum ambient temperature for January and February in this region. Temperature extracted from the timelapse camera was compared to ground-truthed data from a nearby weather station, and a good correlation was observed between both readings within the range of maximum temperatures detected (Dirección General de Aeronáutica Civil de Chile [63]; see S3 Table). By visual inspection it was evident temperatures of 18ºC were also high for chicks as there would be at least one panting chick within the frame when recorded air temperature went above the given threshold.

**Fig 2:**
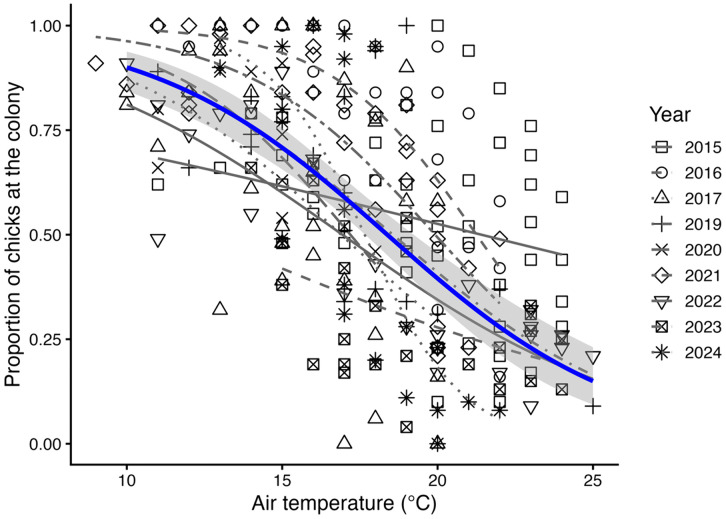
Proportion of chicks at the colony as a function of temperature recorded by time-lapse camera. Proportion of chicks at the colony = (n of chicks at the colony/maximum n of chicks that season), for days reaching ≥ 18°C. Each year indicated with a different symbol depicted in legend. Quasibinomial GLM of the overall proportion of chicks at the colony as a function of temperature and year indicated with black line and 95% confidence intervals in grey (See S4 Table and methods for model details).

The number of chicks at the nest during days reaching temperatures of 18°C and above were counted and presented as proportion of chicks within the colony area of the total counted that season relative to the temperature registered by the camera (Fig 2 and S4 Table). The proportion of chicks at the colony declined significantly with increasing air temperature and differed amongst years (Quasibinomial GLM for proportion of chicks at the colony ~ temperature + year: β = − 0.11 ± 0.02, t = 5.26, P < 0.001 for temperature and β = − 0.05 ± 0.02, t = 2.42, P = 0.016 for year). The temperature at which 50% of chicks remained at the nest was 33ºC in Jan-Feb 2015, 25 ºC in 2016, 20 ºC in 2017, 2019 and in 2020, 26 ºC in 2021 and 19 ºC in 2023 and in 2024. Therefore, we established 20°C as a temperature threshold above which chicks actively moved outside the breeding area presumably to cool down at the beach or under nearby bushes.

### Post-guard correlates

Days with temperatures ≥ 20°C, and number of hours with temperatures ≥ 20°C within the day, were counted for each season. In addition, the number of hours chicks were exposed to high temperatures was estimated as the number of hours ≥ 20°C during post-guard. The number of hot days avoided by chicks was calculated as the total number of days ≥ 20°C between September and March minus the number of days ≥ 20°C during post-guard. A Poisson GLM was generated to evaluate the number of hot days avoided by chicks (total number of hot days (reaching ≥ 20ºC) in the season minus hot days during post-guard) as a function of the date post – guard ended. In addition, a Poisson GLM was generated to evaluate the number of hot hours avoided by chicks (total number of hot hours (≥ 20ºC) in the season minus hot hours during post-guard) as a function of the date post-guard ended (S2 Table). Analyses were done using R software version 4.3.3 [64] and (*pos*, *ggplot2*, *dplyr*, *ggthemes*) packages. Significance was set at P < 0.05. S2 and S4 Tables contain the data used in the analysis.

## Results

### Mortality event of post-guard Gentoo chicks above 20°C temperatures

**Fig 3:**
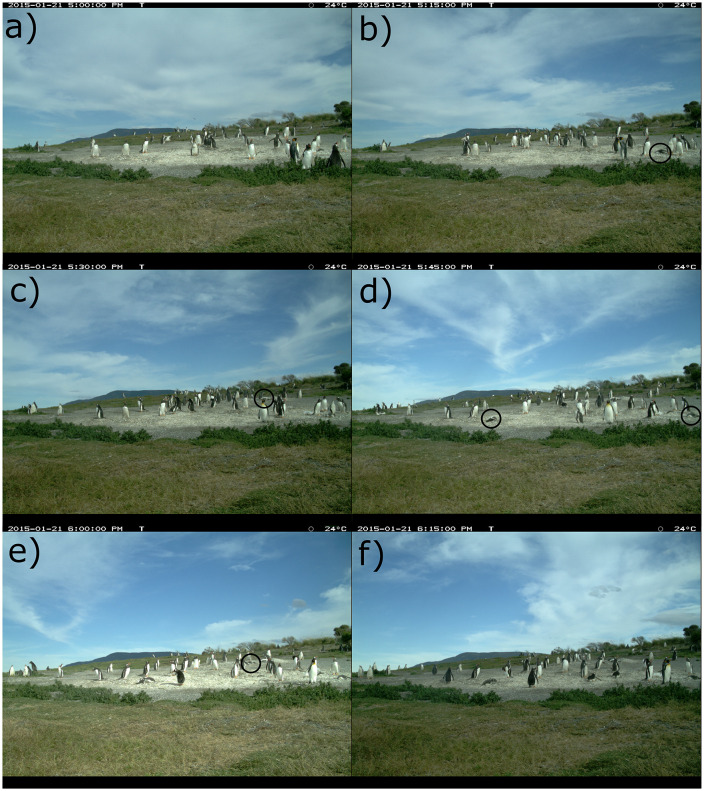
Photographs from time-lapse camera registering the death of chicks on the 21^st^ of January 2015. **(A)** no dead chicks 2015-01-21 5:00:00 PM; **(B)** one dead chick on the left of the image; **(C)** two dead chicks, 2^nd^ in the back; **(D)** four dead chicks, 3^rd^ on the far left, 4^th^ in the center; **(E)** five dead chicks, 5^th^ in the back to the right of the 2^nd^ one. **(F)** five dead chicks 2015-01-21 6:00:00 PM, chicks seemingly panting in all images. Dead chicks indicated with black circle in each photograph. Date and time in legend of photographs as well as temperature recorded by the camera, which was 24°C throughout the sequence. Images are similar but not identical to the original.

During the 2014 season a three-day heatwave (21^st^ to 23^rd^ of January 2015) exposed chicks to a total of 25 h of temperatures above 20°C (see Fig 2), reaching up to 24°C two of those three days. On the first day of the heatwave five of the 32 chicks died within 45 minutes of surpassing 24°C. This event was observed *in situ* and was later corroborated by time-lapse camera images (Fig 3). The cause of death of the Gentoo chicks could not be determined in this study as chicks could only be removed at a later date and carcasses were too decomposed for anatomical inspection. However, we can rule out starvation as the cause of death as their weight relative to size was average for that species and period (they all weighed ≥ 3 kg at the time). Also, no predators were observed and they lacked external injuries or other signs of disease.

### Changes in chick behavior

**Fig 4:**
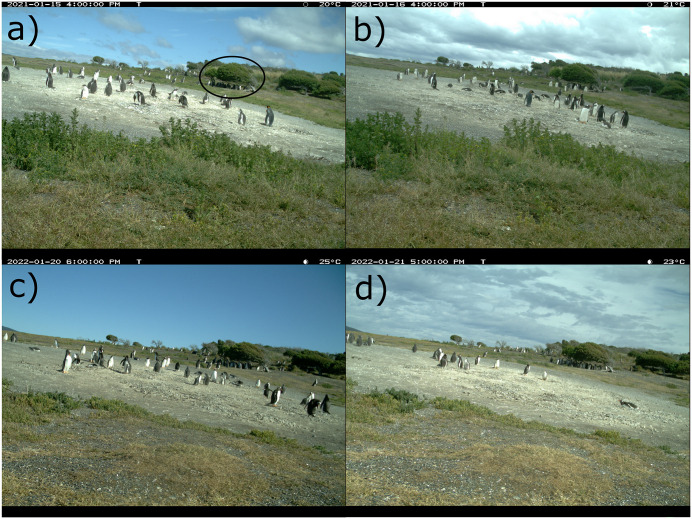
Photographs from time-lapse camera showing chicks under nearby bushes on hot days (temperature ≥ 20°C) in season 2021 in (A) and (B), and in season 2022 in (C) and (D). Nearby bushes indicated with a black circle in image (A). Images are similar but not identical to original.

With temperatures of 18°C and above, which is considered elevated for this region, one or more chicks were seemingly panting (See chicks in Fig 3). In the 2014 season most of the post-guard chicks were seen at the colony during the hottest hours (20–24°C) and death occurred within the colony area. The following seasons, over 50% of post-guard chicks were absent from the colony when temperatures rose above 20°C (Fig 2, methods). In the last five seasons, some of the chicks that were abstent from the colony area were seen using the shade of nearby bushes when temperatures rose above 20°C (Fig 4).

### Timing of post-guard over the years and exposure to high temperatures

**Fig 5:**
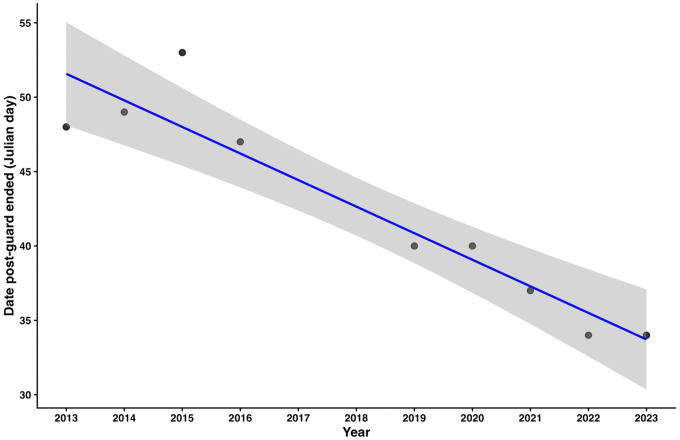
Day of the year post-guard ended each year (2014-2024). Trend of post-guard ending as a function of year in blue with 95% confidence intervals in grey.

The duration of the post-guard period over the studied seasons (2013−2023) was 42 ± 2 days ranging between 39 and 43 days. No temporal trend was detected in post-guard duration (linear model: β = −0.24 ± 0.49 days/year, t= 0.50; P = 0.63). Lay date occurred earlier in successive years shifting from the 20^th^ of October in 2015, to the 26^th^ of September in 2022 (linear model: β = −1.51 ± 0.52 days/year, t = −2.89, P = 0.018, R^2^ = 0.48, n = 10). The end of post-guard also occurred earlier in successive years at a rate of 2 ± 1 days per year (linear model: β = −1.79 ± 0.24 days/year, t = − 7.5 P = 0.001, R^2^ = 0.87, n = 10, Fig 5).

**Fig 6:**
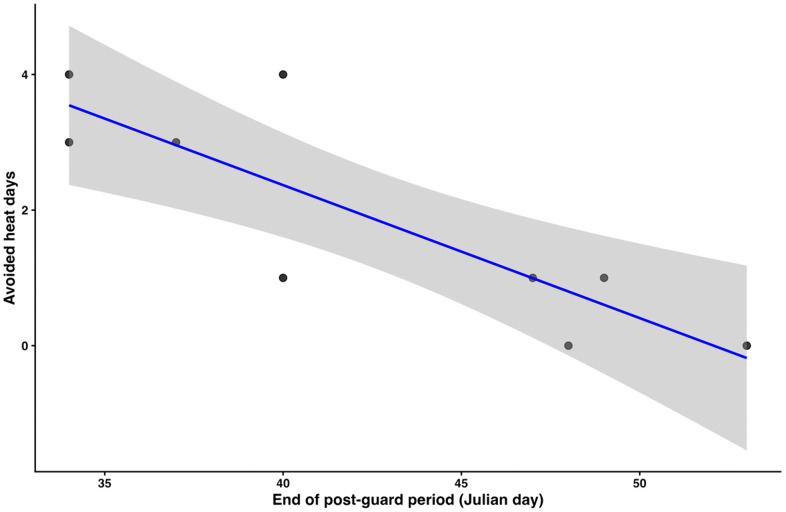
Relationship between the end date of the post-guard period and the number of hot days (≥ 20°C) avoided by chicks. Total number of hot days avoided was calculated as the total number of days with temperatures ≥ 20°C between September and March minus the number of hot days occurring during post-guard period each season. Hot days in February were increasingly avoided by chicks as post-guard ended sooner each year. The fitted trend is shown in blue with 95% confidence intervals in grey.

**Fig 7:**
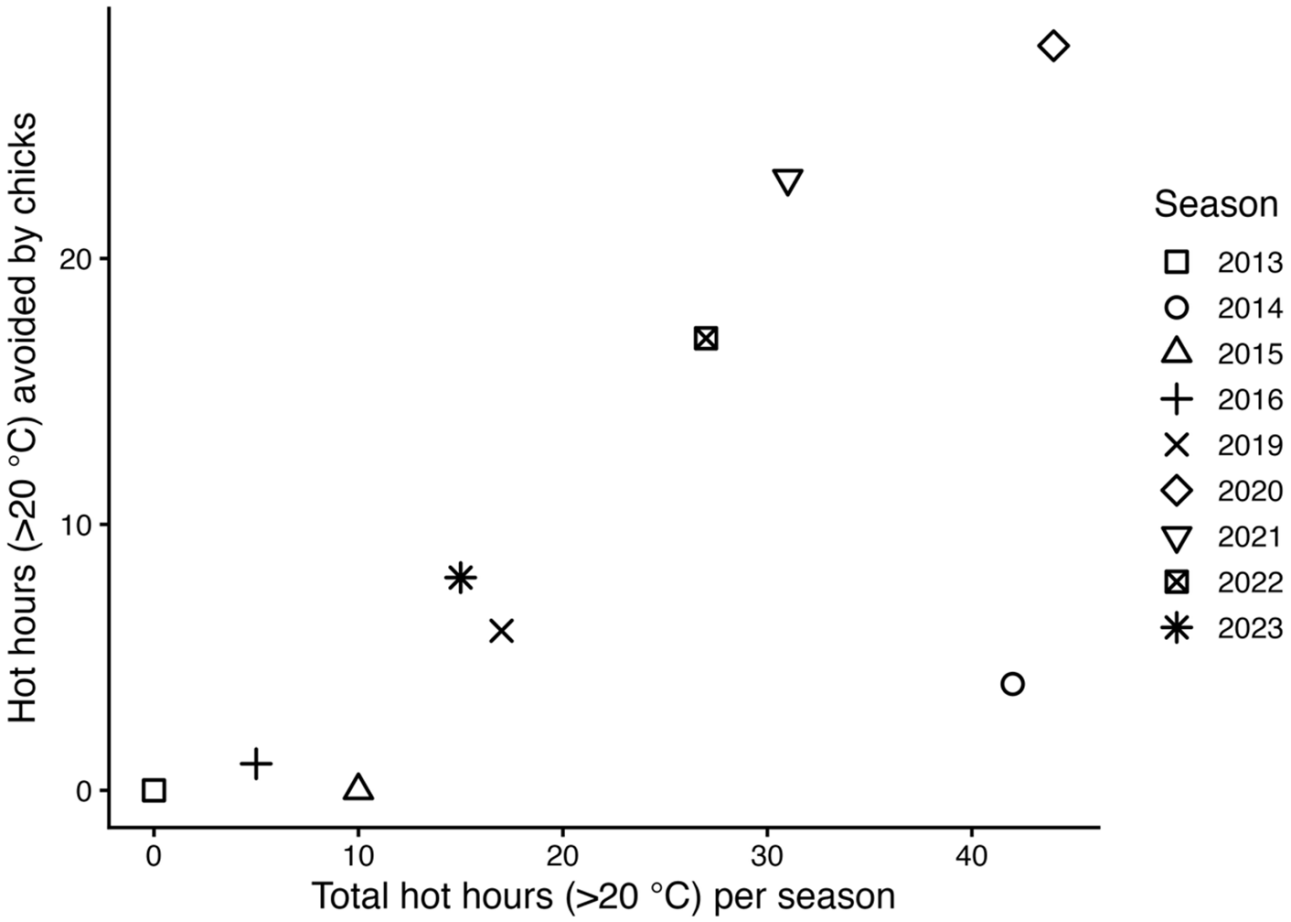
Relationship between the total hot hours (≥ 20°C) in the season and the number of hot hours (≥ 20°C) avoided by chicks during post – guard. Hot hours avoided were calculated as the total number of hours with temperatures≥ 20°C between September and March minus the number of these hours occurring during post-guard. Each symbol represents a breeding season.

Earlier-ending post-guard periods resulted in more hot days avoided by the chicks (Poisson GLM β = −0.13 ± 0.05 heat days avoided/date post-guard ended, z = 2.62, P = 0.008). In earlier seasons post-guard chicks were exposed to most, if not all, the hottest hours of the season (≥ 20°C), and a maximum of 38 hours in 2014 season. In recent years, chicks avoided up to four days of high temperatures (≥ 20°C) when ending the season sooner (Fig 6). In 2014 season only 4 of 42 hot hours were avoided by chicks, but since then, up to 28 of 44 hot hours (season 2020) were avoided by ending post-guard sooner (Fig 7). Since 2021 season, post-guard chicks have been exposed to less than 10 hours of high temperatures and more than 53% of hot hours have been avoided by post-guard chicks as breeding is ending earlier.

## Discussion

Gentoo penguin chicks are sensitive to unusually high temperatures during post-guard. Above 18ºC (90^th^ percentile of the typical temperatures in the area), chicks start panting and above 20ºC they leave the colony for the nearby bushes. Heat stress can result in death to these chicks as observed in a casualty event in January 2015, where 20% of the chicks died over three days, with temperatures reaching 24°C. Unexpectedly, the advancement of the end of the breeding season due to warmer spring conditions (similar to other penguins´ in Antarctica [52]), is giving the chicks the advantage of fledging before they are exposed to most of the hottest summer days. This is a rare example of how a climate-change induced phenological shift aided in the survival of Gentoo chicks to the warmer days.

Climate-induced phenological shifts have been recorded in many species, including seabirds [11,31,52,65,66,72]. Phenological shifts have usually been associated with negative effects on breeding success and survival [67–73]. One mechanism behind this negative effect is suggested to be an increasing mismatch between prey availability and seabird needs while breeding [74,75]. In addition, pathogens and invasive species that can readily adapt and thrive under these changing environmental conditions and put pressure on native species [19,76]. There are only few examples in which warmer spring conditions have had a positive impact on species, such as breeding success of Capercaillie (*Tetrao urogallus*) in Norway [66], or the advancement in phenology linked to an increase in breeding success of king penguins *Aptenodytes patagonicus* in relation to oceanographic characteristics [72]. The present study is a rare example in which the advancement of breeding, due to warmer spring conditions, has had an unexpected positive impact on the breeding outcome of a long-lived seabird that remains at the colony year-round.

At temperatures of 18°C and above, considered above average for the summer in this region, post-guard Gentoo chicks were seen panting, therefore mechanisms to liberate excess heat were already in place at that temperature. The fact that more than 50% of Gentoo chicks were absent from the colony when temperatures went above 20°C during post-guard, except for January 2015, is a good indication that higher temperatures trigger behavioral responses aimed at reducing heat exposure. Other studies have found that temperatures above 20°C increase metabolism to release excess heat in Little Auk *Alle alle* [6], and 22.4°C is the upper critical temperature for the species [77]. Temperatures of 22°C promote behavioral responses such as gular fluttering and standing rather than sitting on the nest by bank cormorant *Phalacrocorax neglectus* [44]. Given chicks are more susceptible to heat as they have fewer behavioral responses than adults, it is likely that temperatures above of 20°C have detrimental effects on Gentoo penguin chicks when they are a few days into post-guard, particularly when they are well fed and metabolically active during feather production and growth [19,28,29]. Sustained high temperatures reaching 24°C during a heat wave, a total of 25h in this case, is presumed to have had a lethal effect on chicks. Knowledge of this upper thermal limit is important to determine the vulnerability of the species in the face of climate change [78]. Caution must be taken as temperature was recorded from a camera a few meters away from the colony and wind chill, humidity and other factors that may have modified the temperature perceived by the penguins were not considered. However, this heat wave was concurrently recorded in Puerto Williams (15 km away) with a historical maximum of 25.2°C on the 22^nd^ of January 2015, described as the highest temperature recorded since 1971 [63].

During this study, end of post-guard occurred sooner at a rate of 2 ± 1 days per year. Post-guard has come forward 23 days from 2013 to 2023, and chicks have avoided up to 27 hours of temperatures above 20°C by fledging sooner in the summer. Gentoo penguins at Martillo Island may be advancing breeding due to earlier warming temperatures or end to snow fall, as has been observed at other colonies [41,52]. These phenological adaptations to climatic conditions at the onset of breeding show the plasticity of the species and their ability to adjust their breeding to Spring weather conditions, as occurs with other resident species [79]. Phenological adaptations might be very important in the survival of highly philopatric species such as this [80]. However, in the future the shift in breeding timing may not be enough to override weather events, therefore if intensity and duration of heat waves continue rising at this location a local extinction may occur.

## Conclusion

Gentoo penguin chicks are highly susceptible to high temperatures during post-guard when they are no longer sheltered by their parents yet are not ready to go to sea. Ambient temperatures of 18°C and above increase thermoregulatory behaviors in chicks and 20°C is the thermal limit above which post-guard chicks tend to move out of the colony area to cool off at the beach or under bushes. In absence of these behavioral responses to heat, sustained temperatures of 24°C were consistent with lethality to chicks. Gentoo penguin plasticity to adjust the timing of breeding is helping chicks avoid the increasing amount of hot summer days on land. The fact that this has occurred without changes to chick productivity means that no phenological mismatch has occurred, however if temperatures above 20°C become more common, this adaptation might not be enough to sustain breeding at this location.

## Supporting information

S1 Table Timing of photographs taken by a Reconyx HC500 Hyperfire time lapse camera monitoring the Gentoo penguin colony at Martillo Island, Tierra del Fuego, Argentina between 2013 and 2024. Frequency of photographs within each interval indicated between brakets. Frequency of photographs varied among years depending on the specific data collected and camera battery life.
(XLSX)

S2 Table Lay date, date post-guard ended, number of hot days and hot hours (≥20ºC) avoided by Gentoo penguin chicks at Martillo Isl. each breeding season (2013–2023). (XLSX)

S3 Table Comparison between maximum temperatures recorded during ambient heat waves (maximum temperatures ≥18ºC, lasting at least three consecutive days) at Puerto Williams meteorological station and maximum temperatures recorded by the time lapse camera those same days. (XLSX)

S4 Table Temperature recorded by time lapse camera at Martillo Isl. and percentage of Gentoo penguin chicks present in the nesting area on days reaching ≥18 °C in 2014–2023 seasons (years 2015–2024). (XLSX)
